# Tick Genome Assembled: New Opportunities for Research on Tick-Host-Pathogen Interactions

**DOI:** 10.3389/fcimb.2016.00103

**Published:** 2016-09-15

**Authors:** José de la Fuente, Robert M. Waterhouse, Daniel E. Sonenshine, R. Michael Roe, Jose M. Ribeiro, David B. Sattelle, Catherine A. Hill

**Affiliations:** ^1^SaBio, Instituto de Investigación en Recursos Cinegéticos IREC-CSIC-UCLM-JCCMCiudad Real, Spain; ^2^Department of Veterinary Pathobiology, Center for Veterinary Health Sciences, Oklahoma State UniversityStillwater, OK, USA; ^3^Department of Genetic Medicine and Development, University of Geneva Medical SchoolGeneva, Switzerland; ^4^Swiss Institute of BioinformaticsGeneva, Switzerland; ^5^Computer Science and Artificial Intelligence Laboratory, Massachusetts Institute of TechnologyCambridge, MA, USA; ^6^Broad Institute of MIT and HarvardCambridge, MA, USA; ^7^Department of Biological Sciences, Old Dominion UniversityNorfolk, VA, USA; ^8^Department of Entomology, North Carolina State UniversityRaleigh, NC, USA; ^9^Laboratory of Malaria and Vector Research, National Institute of Allergy and Infectious DiseasesRockville, MD, USA; ^10^Division of Medicine, University College LondonLondon, UK; ^11^Department of Entomology, Purdue UniversityWest Lafayette, IN, USA

**Keywords:** tick, *Ixodes scapularis*, genomics, proteomics, *Borrelia*, *Anaplasma*, evolution

## Abstract

As tick-borne diseases are on the rise, an international effort resulted in the sequence and assembly of the first genome of a tick vector. This result promotes research on comparative, functional and evolutionary genomics and the study of tick-host-pathogen interactions to improve human, animal and ecosystem health on a global scale.

## Tick-borne diseases: a growing burden for human and animal health worldwide

Ticks are obligate blood-feeding arthropod ectoparasites that are distributed worldwide and one of the most important vectors of pathogens affecting humans and animals (Jongejan and Uilenberg, 2004; de la Fuente et al., 2008). Globally, emerging and re-emerging tick-borne diseases exert an enormous impact on public health (Jones et al., 2008). Urbanization, exploitation of environmental resources and outdoor recreational activities increase human contact with ticks and the transmission of tick-borne pathogens (Gortazar et al., 2014). In addition, tick populations are expanding due to changes in climate (Estrada-Peña et al., 2014) and new tick-borne diseases are emerging (Kosoy et al., 2015; Kernif et al., 2016). However, despite the growing burden that tick-borne diseases represent for human and animal health worldwide, the pace of research in this area has been restricted by the lack of access to a completed tick genome. The recent description of the first tick genome is therefore timely and a spur to future research (Gulia-Nuss et al., 2016).

## The first tick genome sequenced and assembled: results and possibilities

More recently, a global consortium of 93 scientists described the 2.1 Gbp nuclear genome of the black-legged tick, *Ixodes scapularis* (Say) (Gulia-Nuss et al., 2016). This tick species is a vector of pathogens that cause, among others, the emerging diseases Lyme disease [Lyme borreliosis is the most common tick-borne disease in Europe and the U.S. (Centers for Disease Control and Prevention (CDC), 2016; European Centre for Disease Prevention and Control (ECDC), 2016)], human granulocytic anaplasmosis (HGA), babesiosis and tick-borne encephalitis (TBE) (Wormser et al., 2006). The genome of *I. scapularis* Wikel strain was sequenced in a joint effort by the Broad Institute of MIT and Harvard and The J. Craig Venter Institute (JCVI) and funded by the National Institute of Allergy and Infectious Diseases, National Institutes of Health. Annotation for this assembly was produced in a joint effort between JCVI and VectorBase (https://www.vectorbase.org/) with support from The Broad Institute (Gulia-Nuss et al., 2016).

The *I. scapularis* project proved challenging due to the large size and high repeat content of the genome. However, the results show the assembly and description of features associated with ~57% of the genome. As the only assembly available for a tick, the *I. scapularis* genome constitutes an invaluable reference for comparative genomic analyses, including resolution of phylogenetic relationships within the diverse phylum Arthropoda. Analysis of the *I. scapularis* genome revealed new features that may be unique to this organism and with important implications for future research. Highlights include the identification of two new repeat elements, a large-scale gene duplication event that likely occurred ~40 MYA coinciding with tick radiation, gene exon-intron structures more closely resembling that of an ancient protostome/deuterostome ancestor than of extant arthropods examined to date, an expansion of Kunitz domain proteins and other proteins implicated in tick blood feeding, possible remnants of a heme synthesis pathway contrasting with an expansion of heme carrier and storage proteins. Also identified were orthologs for at least 39 invertebrate neuropeptides and neuropeptide receptor genes that are believed or known to regulate tick diuresis, ecdysis, cuticle synthesis, blood feeding and reproduction. The genome contains one of the largest expansions of cytochrome P450 genes known for sequenced arthropods, suggesting potential for rapid development of acaricide resistance in ticks, and it will be important to explore the families of candidate acaricide targets uncovered by genome analyses, the de-orphanisation of which is underway.

The first genome-wide population genomics study suggested genetic variation between ticks from Lyme prevalent northern and mid-western states compared with southern states in the U.S., paving the way for identification of genes tied to vector competence (Gulia-Nuss et al., 2016). The *I. scapularis* genome sequence and annotation also contributed to the characterization of the transcriptome in related tick species such as *I. ricinus* (Genomic Resources Development Consortium et al., 2014; Kotsyfakis et al., 2015), the main vector for tick-borne pathogens of public health importance in Europe. Additional studies that explore the biology of *I. scapularis* and other tick species, and extend genome analyses were described based on the publication of the *I. scapularis* genome sequence (e.g., Cabezas-Cruz et al., 2016; Carr et al., 2016; Egekwu et al., 2016; Grabowski et al., 2016; Van Zee et al., 2016; Zhu et al., 2016). The *I. scapularis* genome also provides a key reference for comparative genomics with other Parasitiformes like the western orchard predatory mite (Hoy et al., 2016), as well as across Chelicerata where large genome sizes often make sequencing and assembly a very challenging undertaking.

**Figure 1:**
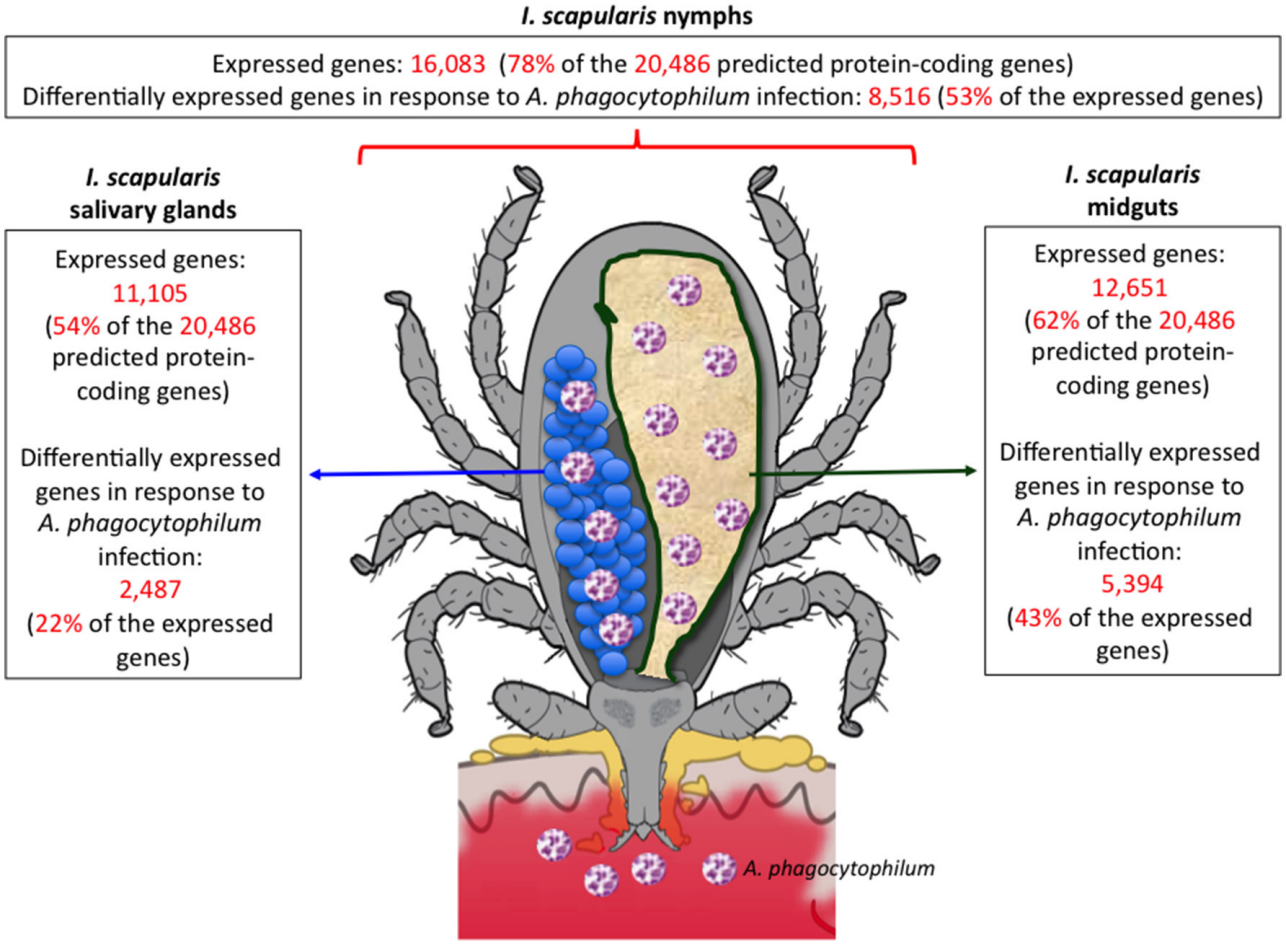
**Example of the advance in the characterization of tick-pathogen interactions based on the sequence and assembly of the ***I. scapularis*** genome**. Transcriptomics data was obtained from Ayllón et al. (2015).

Recent results on the characterization of tick-host and tick-pathogen interactions highlighted the impact of *I. scapularis* genome sequence and assembly on these studies (Figure 1). Transcriptomics, proteomics and metabolomics studies showed the tissue-specific tick response to infection with *Anaplasma phagocytophilum*, the causative agent of HGA (Ayllón et al., 2015; Villar et al., 2015; Alberdi et al., 2016). Complementary proteomics analyses also revealed proteins associated with transmission of *A. phagocytophilum* and the encephalitis-causing Langat virus (Grabowski et al., 2016; Gulia-Nuss et al., 2016). New advances in experimental approaches using omics technologies also boost our knowledge of the tick-host interface (Sojka et al., 2013; Schwarz et al., 2014; Chmelař et al., 2016a,b). Finally, the analysis of the evolution of tick-host-pathogen interactions suggested conflict and cooperation between hosts, vectors and pathogens (de la Fuente et al., 2016a).

## Conclusions and future directions

The features discovered in the *I. scapularis* genome provide insights into parasitic processes unique to ticks, including host “questing,” prolonged feeding, cuticle synthesis, blood meal concentration, novel methods of hemoglobin digestion, heme detoxification, vitellogenesis, reproduction, oviposition, prolonged off-host survival and host-tick-pathogen interactions. The *I. scapularis* gene models will advance research on comparative and functional genomics, while the assembly and physical map will underpin much needed studies of tick genetics. Recent efforts addressed the need for additional tick genomic resources by focusing on tick species relevant for human and animal health (Guerrero et al., 2010; Cramaro et al., 2015). Advances in tick genomics have also facilitated the characterization of the impact of co-infections and microbiome composition on tick vector capacity (Narasimhan and Fikrig, 2015; Vayssier-Taussat et al., 2015). These results greatly improve our understanding of tick biology and will advance research on tick-host-pathogen interactions to develop effective and environmentally friendly measures to control ticks and the many pathogens and parasites they transmit (de la Fuente and Contreras, 2015; Benelli et al., 2016; Carr and Roe, 2016; de la Fuente et al., 2016b; Esteve-Gassent et al., 2016; Kuleš et al., 2016).

## Author contributions

All authors listed, have made substantial, direct and intellectual contribution to the work, and approved it for publication.

## Funding

The National Institutes of Health, the National Institute of Allergy and Infectious Diseases and the U.S. Department of Health and Human Services provided principle funding for the sequence and assembly of the *I. scapularis* genome.

### Conflict of interest statement

The authors declare that the research was conducted in the absence of any commercial or financial relationships that could be construed as a potential conflict of interest.
